# Sex differences in the effect of muscle fatigue on static postural control under different vision and task conditions

**DOI:** 10.1371/journal.pone.0269705

**Published:** 2022-06-22

**Authors:** Donguk Jo, Maya Pannetier, Sophie Drouin, Sarah Bassil, Caroline Matte, Martin Bilodeau

**Affiliations:** 1 School of Rehabilitation Sciences, Faculty of Health Sciences, University of Ottawa, Ottawa, Ontario, Canada; 2 Aging and Movement Laboratory, Bruyère Research Institute, Ottawa, Ontario, Canada; 3 School of Human Kinetics, Faculty of Health Sciences, University of Ottawa, Ottawa, Ontario, Canada; Universite de Caen Normandie, FRANCE

## Abstract

The main aim of this study was to compare the effects of ankle plantar flexors fatigue on postural control between healthy young adult males and females. The secondary aim was to determine the effects of vision on the fatigue-induced postural changes. Ten healthy young males and nine females were asked to perform quiet standing (QS) and standing forward lean (FL) tasks with eyes open (EO) and closed (EC) before and immediately following exercise, and throughout a 15-min recovery period. A sustained isometric exercise of ankle plantar flexors was performed until participants were no longer able to maintain a target torque of 50% of maximal voluntary isometric contraction (MVIC). Mean anteroposterior (AP) and mediolateral (ML) positions of the center of pressure (COP), mean COP sway velocity, and 95% ellipse area of COP sway were measured. Ankle plantar flexors fatigue had significant effects on all dependent variables, except for sway area. A fatigue X sex interaction was found for sway velocity with the most challenging task condition (FL-EC), where males showed a significant increase in sway velocity up to 15 min following exercise, whereas females did not. Fatigue X vision interactions for AP position were also found, with the withdrawal of vision leading to a greater backward shift during recovery for both the QS (5 to 15 min) and FL (5 to 10 min) tasks. Our findings suggest the use of different postural control strategies with ankle fatigue between males and females, and also a contribution of vision to compensate for fatigue-induced instability that is not dependent on task difficulty.

## Introduction

Postural stability is regarded as the capability to control the center of mass with respect to the base of support [1]. To maintain postural stability, the central nervous system must integrate sensory information from the visual, vestibular, and somatosensory systems [2]. The central nervous system must also use this information selectively to produce complex motor responses (i.e., timing, direction, and magnitude), appropriate to the characteristics of the postural stability disturbances of the surrounding environment [2].

Acute exercise-induced muscle fatigue can affect sensory information and motor commands required to regulate postural control [3]. This is supported by review studies [4, 5], which conclude that muscle fatigue leads to modifications in the peripheral proprioceptive system and central processing of sensory inputs [4], in addition to the decrements in force production capacity [5]. Fatigue of postural muscles can thus alter or impair postural control performance. Many studies, for example, have found changes in static postural control following strenuous resistance exercise (isometric or isokinetic) of postural muscles (trunk, hip, knee, and ankle) in healthy adults [6–13]. In particular, ankle plantar flexors fatigue induced by such strenuous exercise can lead to significant changes in postural control during upright standing [8, 12, 14, 15]. This can be attributed to the significant role of ankle plantar flexors in regulating active ankle stiffness to maintain postural stability during upright standing [16].

The rate and extent of fatigue can be affected by sex-related physiological differences [17, 18], such as: a greater proportion of fatigue-resistant muscle fibers within skeletal muscles [17], lesser neural drive to the muscle [19] and greater oxidative metabolic capacity [20] in females compared to males. Such differences could lead to greater fatigability in males compared with females, especially when performing low-to-moderate isometric exercise [17, 18], and this could lead to greater alterations in postural control in males during certain tasks. Only a limited number of studies have looked at sex differences with regards to the effect of fatiguing postural muscles on stability. For example, greater COP sway displacements [21], increased COP velocity (only in males) during one-leg standing [22], and reduced leg-reaching distance [23] have been observed in males compared with females. These findings suggest different postural control strategies between males and females with fatigue. Particularly, an ankle strategy, ankle joint control (or movements) to maintain stability, could be a primary contributor to such difference (e.g., during quiet standing, see [24]). However, the effect of sex on fatigue-related change in postural control is still unclear, including in challenging tasks, such as when leaning forward close to the limit of stability.

Furthermore, vision may affect the extent of fatigue-related changes in postural control depending on the postural task. A few studies [7, 25, 26] where fatigue of ankle plantar flexors was induced, found greater impairments in postural stability during one-leg standing with eyes closed (EC) compared with eyes open (EO). The withdrawal of vision, however, tended not to lead to greater fatigue-induced postural control changes with relatively easier postural tasks (e.g., quiet standing on both legs) (see [14, 27] for example). These contrasting findings suggest that visual information may compensate for fatigue-induced impairments in postural stability depending on task difficulty.

The present study was carried out to provide further information on potential sex differences concerning the effect of ankle fatigue on postural control, and whether such effect could be dependent on postural task difficulty. Given the primary role of ankle muscles in standing stability [16], such information may contribute to basic knowledge of sex differences in injury risks during physically demanding tasks/sports that requires standing postural control in healthy young adults [28–32]. Therefore, the main aim of this study was to determine sex differences in postural control changes generated by fatigue of ankle plantar flexors in healthy young adults in two postural tasks of varying difficulty: a quiet standing (QS) task and a forward lean (FL) task. The secondary aim was to determine the effects of vision (EO vs. EC) on postural changes with fatigue according to task difficulty. It was hypothesized that males would show greater fatigue-induced alterations in postural control compared with females, and such results would be more pronounced under relatively challenging task conditions (e.g., FL-EC) compared to lesser challenging conditions (e.g., QS-EO). Also, the withdrawal of vision would lead to greater fatigue-induced postural changes, which would be more pronounced during the FL task.

## Methods

### Participants

Ten healthy young males and nine healthy young females volunteered for this study. Participants had no disorders or musculoskeletal injuries (e.g., ankle injury and instability) in the past year that could affect postural control, as confirmed verbally. The study was approved by the research ethics boards of the University of Ottawa (# H11-14-23) and Bruyère Continuing Care (#M16-08-038). All participants were informed of all procedures, and their signed consent was obtained prior to participation in this study.

### Experimental procedure

**Fig 1:**
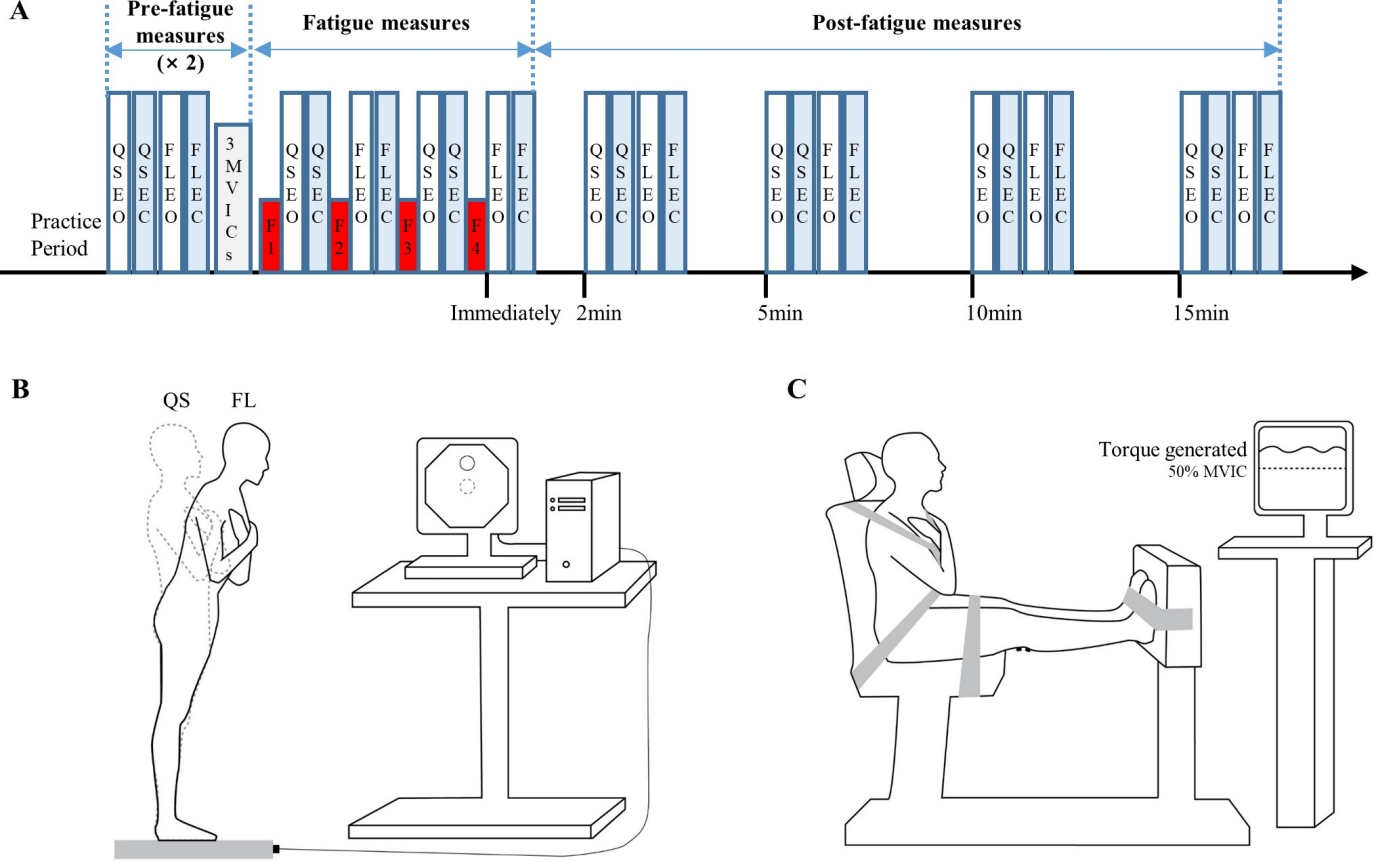
Schematics of the experimental protocol. The schematics include experimental procedures (A), and experimental setup for the postural tasks (QS vs. FL) performed on a force platform with the representation of the COP on a computer screen (B) and sustained isometric exercise of ankle plantar flexors on a dynamometer (C). QS: quiet standing; FL: standing forward lean task; EO: eyes open; EC: eyes closed; F1-F4: four submaximal fatiguing isometric contractions of ankle plantar flexors; MVIC: maximal voluntary isometric contraction; circle: 70% anterior limit of stability (LOS) target for the FL task; dotted circle: 0% LOS target for the QS task.

A schematic of the experimental procedure is shown in Fig 1A. All participants volunteered for a single session, which was held during regular daytime hours (between 9am and 5pm). Participants were asked to avoid strenuous or prolonged physical activity for 24 hours before their session. Each participant was first asked to perform two sets of four postural tasks (QS-EO, QS-EC, FL-EO, and FL-EC) barefoot on a force platform. Before these pre-fatigue measures, all participants were allowed to practice each of the task conditions twice, with instructions to keep their body straight (no flexion at the hips) limiting the movement to the ankles when leaning forward and without lifting the heels off the platform. They then performed three bilateral maximal voluntary isometric contractions (MVICs) in ankle plantarflexion with a 2-min interval between each, from which the highest torque was used to set the target torque for the fatiguing exercise to be performed after the MVIC (see below for details). In order to ensure an adequate level of fatigue when testing the four different postural conditions, only two trials were tested (e.g., QS-EO/QS-EC or FL-EO/FL-EC) after the fatiguing exercise, which was repeated a total of four times. Each of the four postural tasks was then assessed at 2, 5, 10, and 15 min following the end of the fourth exercise protocol. The order of the postural tasks was counterbalanced across participants.

### Postural tasks

Fig 1B shows a schematic of the experimental setup for the FL and QS tasks. Subjects were asked to align the tip of their toes with a line drawn on the force platform located 17 cm from the center of the platform. For the FL task, the limits of stability (LOS) were first obtained by measuring the maximum excursion of the COP while participants lean the whole body in a standing position (feet together and wearing no shoes) as far as possible forward, backward, and side-to-side on a force platform without lifting the heels and limiting the movement to the ankle. Afterward, participants were asked to track a target (small circle) with a representation of their COP on a computer screen placed in front of them. When the target moved upward to a position representing 70% of the anterior LOS on the force platform, the participants were required to maintain the 70% LOS target as closely as possible for 20 s with EO or EC. For the QS task, they were asked to maintain an initial target (representing 0% LOS of all directions) as closely as possible for 20 s while standing with feet together with EO or EC. For the EC condition, participants were first asked to match the 0% or 70% LOS target with EO and once they felt comfortable, to close their eyes and maintain the memorized target for the recorded 30-s trial.

### Fatiguing exercise

Fig 1C shows a schematic of the experimental setup for the fatiguing exercise. Participants sat on the chair of a dynamometer apparatus (Biodex medical systems, Shirley, USA) with their legs straight forward and both feet placed on a footplate (aligning the external malleoli with the center of rotation of dynamometer). Their trunk, thighs, and feet were secured with non-elastic straps. For the fatigue task, they were asked to exert a plantarflexion isometric torque (with both feet) corresponding to 50% MVIC and represented by a trace and target on a computer screen located beside them. The fatiguing protocol stopped when participants no longer produced and maintained the target torque during three consecutive seconds. Verbal encouragement was given throughout.

### Data analysis

COP data were recorded using an AMTI AcuGait force platform (Watertown, MA) at a 25 Hz sampling rate. The following variables were obtained from the Balance Trainer Software (version 1.05.02) from the unfiltered data: mean anteroposterior (AP) and mediolateral (ML) COP position (cm; average AP and ML sway displacement from the platform center = $\frac{1}{n}*\sum_{i=1}^{n}x_{i}$ and $\frac{1}{n}*\sum_{i=1}^{n}y_{i}$, respectively), mean COP sway velocity (cm/s; path length per unit time = $\frac{l_{path}}{t}$), and the 95% ellipse area of COP sway (cm^2^; area of the 95^th^ percentile ellipse = $\pi *\sqrt{F*(x_{sd}^{2}+y_{sd}^{2}+D})*\sqrt{F*(x_{sd}^{2}+y_{sd}^{2}-D})$): $l_{path}=\sum_{i=2}^{n}\sqrt{{\left(x_{i}-x_{i-1}\right)}^{2}+{\left(y_{i}-y_{i-1}\right)}^{2}}$; $D=\sqrt{{\left(x_{sd}^{2}+y_{sd}^{2}\right)}^{2}-4*(x_{sd}^{2}*y_{sd}^{2})-\sigma_{xy}}$; and F statistics = 3.00.

### Statistical analysis

The minimum sample size required was calculated using G* power (version 3.1.9.4) and shown to be at least 7 participants per group, with a power of 0.80, an effect size of 0.68, and alpha of 0.05. The effect size was estimated from existing data, with respect to sex differences in AP sway displacement changes produced by ankle plantar flexors fatigue [21].

Independent t-tests were used to compare demographic information of participants (age, height, weight, and BMI) and MVIC torques between males and females. A Pearsons’ correlation analysis was conducted to determine the association between each of the four COP variables (mean AP and ML positions, sway velocity, and sway area) at pre-exercise and each of three anthropometric variables (height, weight, and BMI), not only overall but also for males and females separately. These correlations were conducted to determine whether sex differences in the anthropometric variables (e.g., smaller height and lesser weight in females compared to males) would be beneficial for controlling standing stability in females, as suggested in previous studies [3, 23, 33, 34].

Separate three-way ANOVAs with one between-subject and two within-subject factors were used to assess the effects of fatigue [pre-fatigue vs. fatigue (immediately) vs. recovery (2, 5, 10, 15 min)], vision [EO vs. EC], sex (males and females) and their interactions for each of the QS and FL tasks on the four COP variables (mean AP and ML positions, sway velocity, and sway area). If Mauchly’s test of Sphericity (within-subjects effect) was significant (≤ 0.05), the Greenhouse–Geisser correction was used to determine significance. When the ANOVAs depicted significance, *post-hoc* t-tests with Bonferroni corrections were performed. In order to assess the impacts of the anthropometric variables on the COP variables according to fatigue and related interactions, the ANOVAs were performed and compared with the four types of COP data: 1) non-normalized, and normalized by 2) height, 3) weight and 4) BMI.

## Results

### Baseline information and measures

Height, weight, and MVIC torque were found to be significantly greater in males compared with females, while age and BMI were not (see details in Table 1). There were significant moderate-to-high negative correlations between both body height and weight and mean AP COP position (overall, as well as in females; see details in Table 2). No significant correlation was noted for the other COP variables (overall and in both females and males), except for sway area, with only males showing a significant correlation with height (r = 0.641; CI = 0.158–0.947; and p = 0.046) for the QS-EC task.

**Table 1:** Sex differences in demographics and MVIC.

	Mean (SD)			*P*-values
	Overall	Males	Females	
Age (years)	25.3 (3.3)	25.6 (3.9)	25.0 (2.6)	0.702
Height (cm)	171.9 (10.1)	178.7 (7.6)	164.3 (6.5)	**<0.001**
Weight (kg)	75.2 (16.7)	85.6 (15.4)	63.5 (8.2)	**0.001**
BMI (kg/m^2^)	25.6 (4.6)	26.9 (5.2)	23.5 (2.7)	0.091
MVIC torque (Nm)	219.4 (53.3)	245.7 (51.1)	190.1 (39.8)	**0.017**

BMI: body mass index = weight/(height)^2^; MVIC: maximal voluntary isometric contraction.

**Table 2:** Results of Pearsons’ correlation analysis of mean AP COP position with body height and weight.

Group	COP variable	Tasks	Height			Weight		
			r	95% CI of r	P-value	r	95% CI of r	P-value
Overall (n = 19)	AP COP mean position	QSEO	-0.629**	-0.81 –-0.39	0.004	-0.514*	-0.83 –-0.23	0.024
		QSEC	-0.547*	-0.79 –-0.25	0.015	-0.495*	-0.82 –-0.17	0.031
		FLEO	-0.414	-0.68 –-0.11	0.078	**-**0.439	-0.80 –-0.12	0.060
		FLEC	-0.533*	-0.78 –-0.21	0.019	-0.526*	-0.83 –-0.24	0.021
Males (n = 10)	AP COP mean position	QSEO	-0.192	-0.60–0.47	0.595	0.246	-0.34–0.80	0.494
		QSEC	0.151	-0.53–0.77	0.676	0.336	-0.20–0.81	0.342
		FLEO	0.392	-0.25–0.90	0.263	0.367	-0.35–0.76	0.296
		FLEC	0.386	-0.28–0.92	0.271	0.296	-0.47–0.70	0.406
Females (n = 9)	AP COP mean position	QSEO	-0.406	-0.81–0.26	0.278	-0.749*	-0.97 –-0.28	0.020
		QSEC	-0.458	-0.86–0.17	0.215	-0.797*	-0.96 –-0.58	0.010
		FLEO	-0.250	-0.74–0.45	0.517	-0.691*	-0.96–0.12	0.039
		FLEC	-0.388	-0.83–0.26	0.302	-0.637	-0.97–0.02	0.065

r: correlation coefficient; CI: confidence interval.

### Fatiguing bouts duration

There were no significant sex differences in the duration of fatigue bouts, even though males presented with somewhat longer mean times to failure (± standard deviation) for the first (159.8 ± 69.7 s), second (106. 8 ± 38. 3 s), third (95.0 ± 29.9 s), and fourth exercise bouts (94.3 ± 38.3 s), compared with females (97.1 ± 21.3 s, 83.9 ± 18.6 s, 77.5 ± 24.5 s, and 74.4 ± 16.7 s, respectively).

### COP sway parameters

Table 3 shows a summary of statistical results (ANOVAs) for non-normalized COP sway parameters for each of the QS and FL tasks. These results were not different from those with the COP data normalized by height or body weight, except for weight-normalized data eliminating a significant sex X fatigue interaction (shown in Table 3) on mean COP sway velocity during the FL task (F = 1.729, p = 0.18); this interaction elimination was described in the discussion section (see below). Results are thus reported only for the non-normalized data and do not focus on the effect of vision alone, as this was not the aim of our study.

**Table 3:** Summary of statistical results (ANOVAs) of COP sway parameters. These results depict the effects of fatigue (time), sex, vision, and fatigue-related interactions during quiet standing (QS) and standing forward leaning (FL) tasks. The results are shown for the COP data that are not normalized by anthropometric variables (weight, height, and BMI).

Type of task	Effects	COP sway parameters							
		*AP position*		*ML position*		*velocity*		*Area*	
		*F*	*P*	*F*	*P*	*F*	*p*	*F*	*P*
*QS*	*Time*	9.810	**<0.001**	4.150	**0.002**	3.404	**0.007**	2.679	**0.027**
	*Sex*	13.93	**0.002**	1.336	0.264	0.229	0.638	1.828	0.194
	*Vision*	4.104	0.059	0.022	0.883	95.63	**<0.001**	179.5	**<0.001**
	*Time x sex*	1.023	0.392	0.879	0.479	1.605	0.167	0.337	0.889
	*Time x vision*	9.167	**<0.001**	2.273	0.054	1.398	0.233	1.537	0.187
	*Time x vision x sex*	1.020	0.395	1.214	0.310	2.181	0.064	0.580	0.715
*FL*	*Time*	4.219	**0.002**	2.766	**0.023**	3.038	**0.014**	1.430	0.222
	*Sex*	14.96	**0.001**	1.385	0.255	0.317	0.581	0.542	0.472
	*Vision*	35.82	**<0.001**	0.085	0.774	87.90	**<0.001**	89.76	**<0.001**
	*Time x sex*	1.988	0.089	0.819	0.539	2.567	**0.033**	1.140	0.346
	*Time x vision*	3.921	**0.003**	1.664	0.152	1.862	0.110	0.624	0.682
	*Time x vision x sex*	2.298	0.052	1.623	0.163	1.052	0.393	0.820	0.539

BMI: body mass index = weight/(height)^2^.

### Mean AP COP position

**Fig 2:**
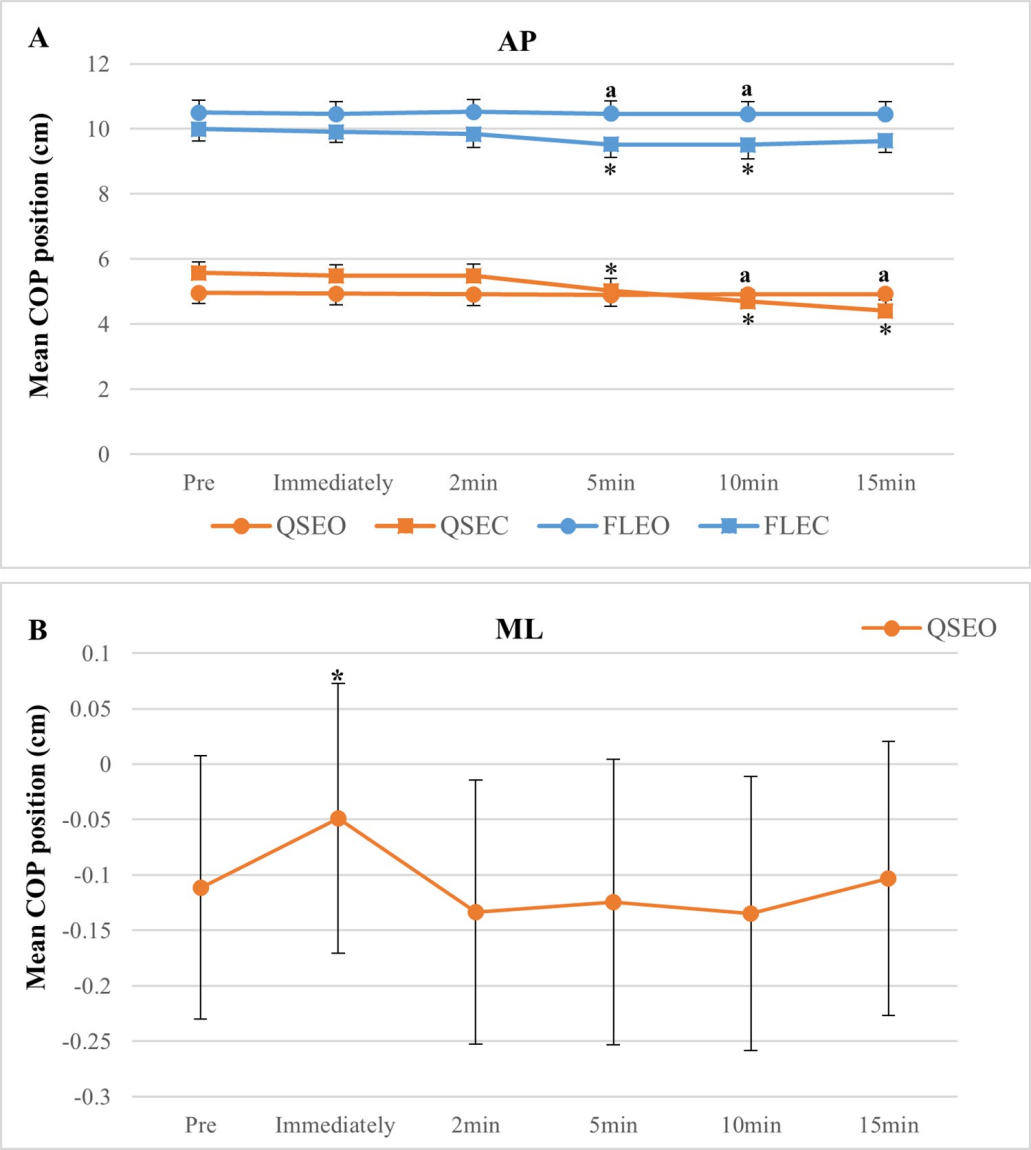
Mean anteroposterior (AP) and mediolateral (ML) COP position. Mean AP and ML COP positions shown before and immediately following exercise, and throughout the recovery period (2, 5, 10, and 15 min). Panel A shows the AP position during both quiet standing (QS) and standing forward leaning (FL) tasks with eyes open (EO) and closed (EC). B represents ML position during QS-EO. * = fatigue effects (*p* ≤ 0. 05, compared to pre-exercise value), and a = effects of fatigue X vision (*p* ≤ 0. 05, in comparing EO to EC).

The effects of sex, fatigue, and a fatigue X vision interaction were found for mean AP COP position for both QS and FL (Table 3). The sex effect was due to a greater mean anterior COP position during all task conditions (QS-EO: ~47%; QS-EC: ~62%; FL-EO: ~21%; FL-EC: ~32%) in females compared to males. The mean AP COP position showed a significant backward shift (mean COP position decreased in value, which is associated with a more posterior location on the force platform) following exercise during QS-EC (~20%; 5min: *p =* 0.03, 10min: *p* = 0.005, and 15min: *p* = <0.001) and FL-EC (~4.9%; 5min: *p =* 0.04 and 10min: *p* = 0.001) (Fig 2A). Fatigue X vision interactions were due to greater backward shifts in mean AP position for both QS (10 min: *p =* 0.03 and 15 min: *p* = <0.001) and FL (5 min: *p =* 0.04 and 10 min: *p =* 0.005) with EC (~20%), as compared with EO (~2%) (Fig 2A).

### Mean ML COP position

A fatigue effect on mean ML COP position was due to a rightward displacement (56%) from pre to immediately post-exercise during QS-EO (*p* = 0.03) (Fig 2B).

### Mean COP sway velocity

**Fig 3:**
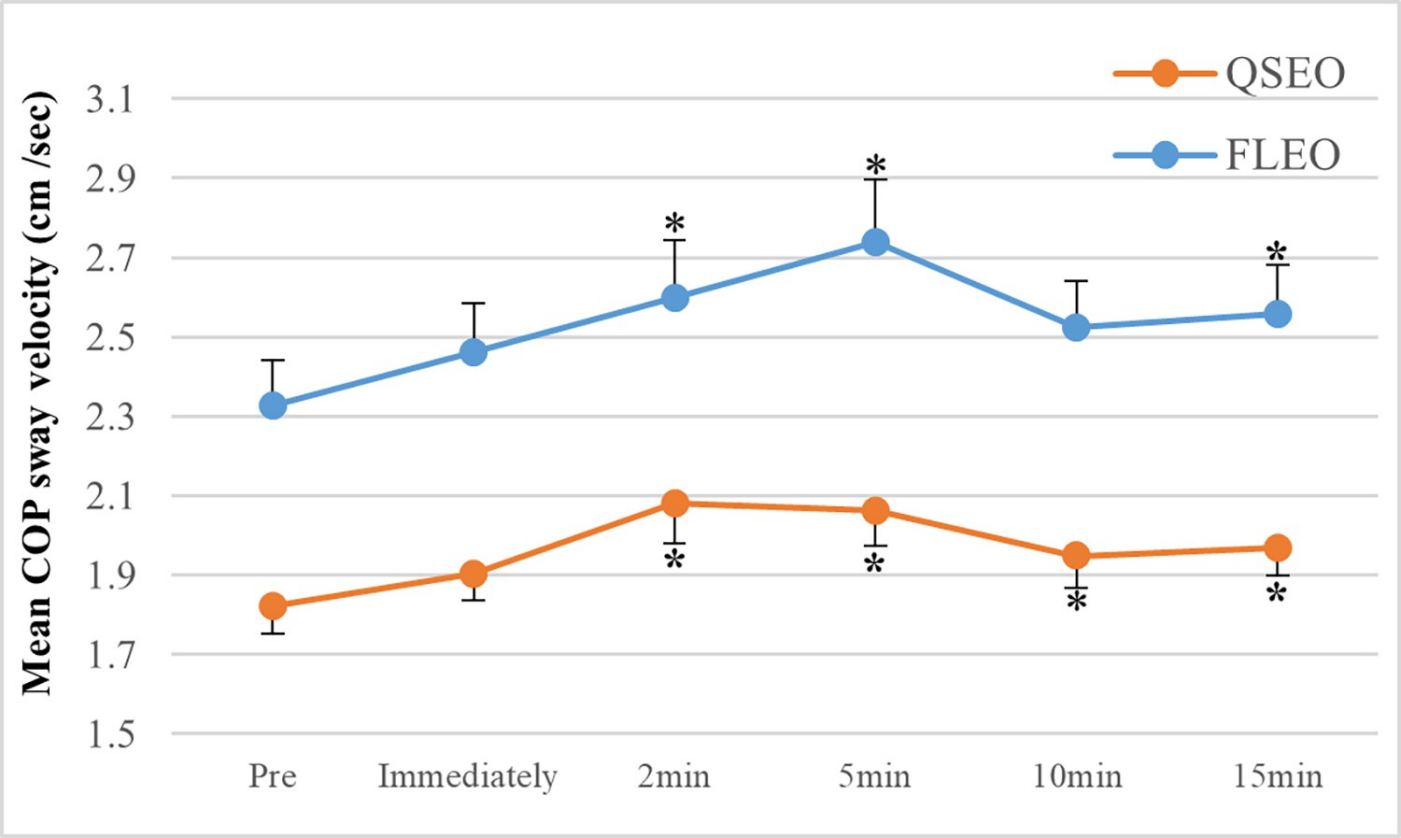
Mean COP sway velocity. Mean COP velocity shown before and immediately following exercise, and throughout the recovery period (2, 5, 10, and 15 min) for quiet standing (QS) and standing forward leaning (FL) tasks with eye open (EO). * = significant fatigue effects (*p* ≤ 0. 05, compared to pre-exercise) on sway velocity.

Fatigue effects and a sex X fatigue interaction (FL) were found for mean COP sway velocity (Table 3). The fatigue effects were due to significant increases in sway velocity following exercise for both QS-EO (~14%; 2 min: *p =* 0.02, 5 min: *p =* 0.01, 10min: *p =* 0.05, and 15 min: *p =* 0.04) and FL-EO (~18%; 2min: *p =* 0.05, 5min: *p =* 0.02 and 15min: *p =* 0.03) (Fig 3). Post hoc tests did not show such significant increases for the EC condition.

**Fig 4:**
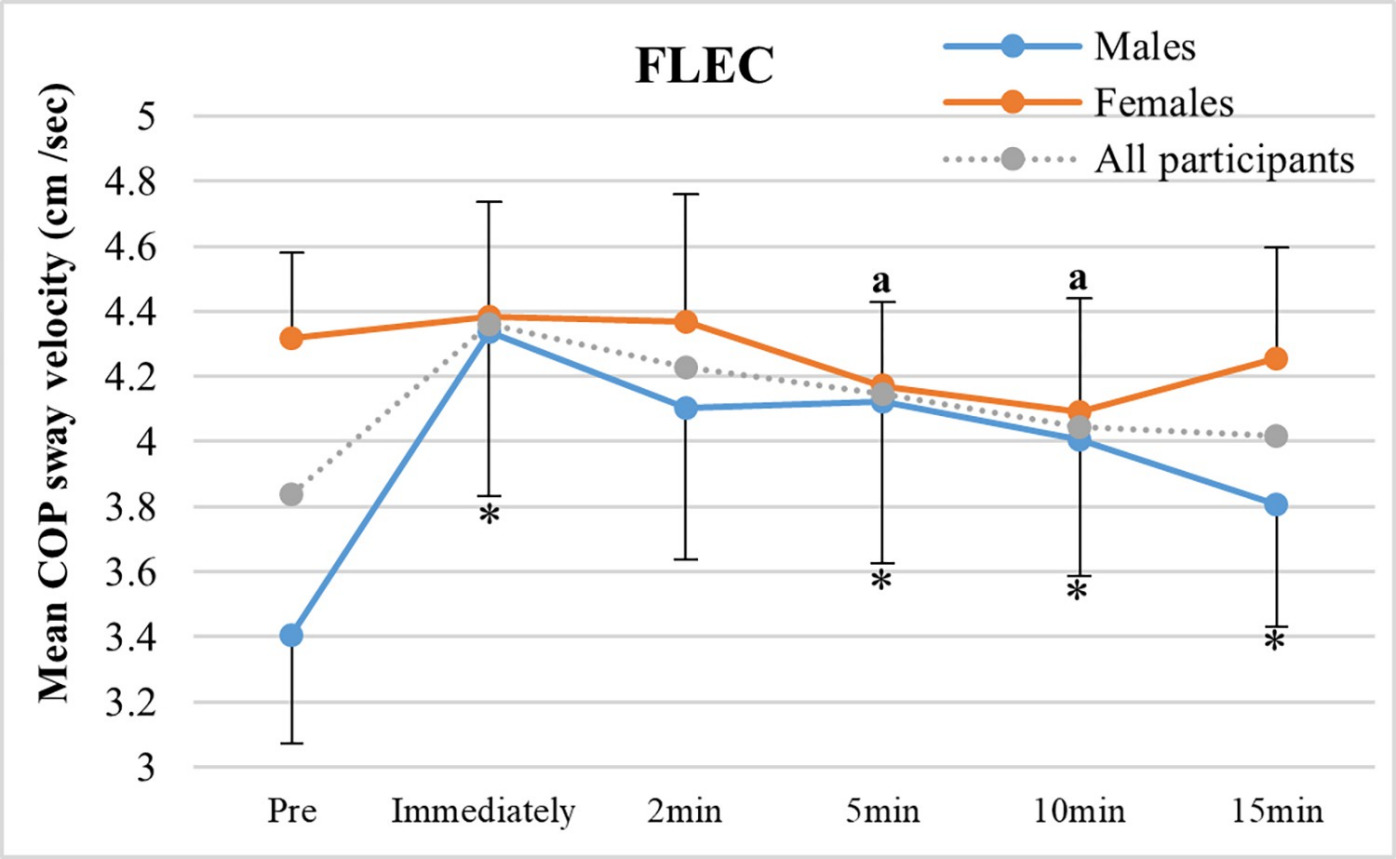
Sex differences in fatigue effects on mean COP sway velocity. Mean COP velocity shown before, immediately following exercise, and throughout the recovery period (2, 5, 10, and 15 min) in males and females during the standing forward leaning task with eyes closed (FL-EC). * = fatigue effects for males and females (*p* ≤ 0.05, compared to pre-exercise), and a = sex X fatigue interaction (p ≤ 0. 05, in comparing percentage changes from pre to post-exercise between males and females).

A sex X fatigue interaction (5min: *p* = 0.05 and 10min: *p* = 0.02) was found during FL-EC, where males had significant and prolonged increases (~18%; immediately: *p* = 0.04, 5min: *p* = 0.02, 10 min: p = 0.002) in sway velocity following exercise, whereas females showed no significant change with fatigue and recovery (Fig 4).

### 95% ellipse of COP sway area

A significant fatigue effect was found for sway area for the QS task. However, none of the *post-hoc* tests reached significance.

## Discussion

The main aim of this study was to compare the effects of ankle plantar flexors fatigue on static postural control between healthy young males and females in tasks with varying levels of difficulty. Our findings showed a sex X fatigue interaction for COP sway velocity during the most challenging task (FL-EC), where males presented with a significant and prolonged increase in velocity, whereas females did not. This is partly consistent with our first hypothesis, which stated that males would show greater fatigue-induced postural control alterations compared with females, and such changes would be more pronounced under challenging task conditions. We also found vision X fatigue interactions for mean AP COP position during both QS and FL tasks, which were due to greater fatigue-induced backward shifts with EC compared with EO. This is partly in accordance with the secondary hypothesis, where it was proposed that the withdrawal of vision would lead to greater postural changes with fatigue, which would be more pronounced during the FL task.

### Fatigue effects on postural control

Ankle plantar flexors fatigue led to changes in mean COP position and velocity. A gradual posterior shift in mean COP position (significant from 5 to 15 min) following exercise was observed during the no-vision tasks (QS-EC and FL-EC). This is in accordance with previous findings, but for unipedal standing tasks [7, 35]. For example, Boyas and colleagues [35] observed a gradual and prolonged (at least 8 min) backward shift of the COP during one-leg standing after fatigue of ankle plantar flexors. This posterior shift could be a compensatory consequence in response to declines or changes in the force-producing capability and proprioception of fatigued ankle plantar flexors [3]. Particularly, the contribution of proprioception is supported by the observation that vibration of fatigued ankle plantar flexors does not significantly produce a further increase in postural sway during upright standing [36, 37], suggesting a lesser reliance on proprioception of the fatigued ankle muscles for regulating postural control during standing. Interestingly, the fatigue-induced posterior shift (pre- to post-exercise) in the mean AP COP position with EC was more pronounced for the relatively lesser challenging task (QS; ~21%) compared to the FL task (~4%). This may be explained by the initial position of the COP for the FL task with EC which was not as far forward compared with EO at pre-exercise. This was opposite to the QS task where the COP was positioned more forward with EC compared to EO at pre-exercise (Fig 2). The less forward COP position during the FL task with EC compared to EO at pre-exercise may be explained by a perceived threat of a forward fall when leaning the body forward to 70% of the LOS. This may be somewhat similar to the COP displacement seen with height-induced threats, which shows a shift away from the platform’s edge (less anterior position) during quiet standing at height compared to relatively lower height or on the ground, possibly due to increased attention to postural control [38].

Interestingly, the significant backward shift was not observed before 5 min post-exercise. Sensory systems likely not affected by fatigue (e.g., vestibular and neck somatosensory inputs, see [39]) could play a initial compensatory role for the reduced ability of fatigued ankle plantar flexors to maintain stability. As well, an initial compensation by other muscle groups, such as ankle mediolateral stabilizers/dorsiflexors, toe flexors or more proximal muscle groups of the knee/hip/back as suggested in previous studies [35, 40, 41], could also be involved. For example, an increase in the contribution of proximal muscles and joint movements (e.g., hip) depending on the extent of ankle fatigue, could explain the backward shift in COP. The aforementioned study by Boyas et al. [35], showed greater increases in hip flexion of supporting and free legs immediately (8%) following exercise compared to during recovery (5%), which were accompanied by a smaller backward shift with fatigue compared with recovery. Such compensation could have taken place in the present study, such that changes in AP hip control may have eliminated or minimized the backward shift of the COP with fatigue of ankle plantar flexors, but mostly immediately after fatigue.

In addition, a fatigue-induced shift of the mean ML COP position to the right was found during the QS-EO task in the present study (shift to a more positive value indicating a shift to the right; Fig 2B), possibly suggesting a fatigue-induced asymmetry of posture. This would be consistent with the results of a recent study showing an increased asymmetry index between preferred and non-preferred legs (based on muscle activation during quiet standing) with ankle and hip muscle fatigue, possibly indicating a shift in the control of posture towards the preferred leg [42]. This suggests that the contribution of a more reliable leg to control posture could increase with fatigue. The right shift of the ML position during QS-EO in the present study could be thus explained by the fact that the right leg is generally observed to be dominant in young adults (e.g., more than 95% in both males and females, see [43]); although we did not assess leg dominance in our participants.

Post hoc tests revealed that significant fatigue-induced increases in sway velocity were found only during the QS-EO and FL-EO tasks. An increase in sway velocity is consistent with previous findings [7, 15, 35, 44]. It has been suggested that increases in sway velocity and corresponding sway frequency could be the result of an increase in ankle stiffness to maintain stability [45]. The lack of a significant increase in sway velocity immediately following exercise (Fig 3) may also be explained by the aforementioned compensatory strategies at proximal joints with fatigue, which could minimize the effect of an increase in ankle stiffness with fatigue of ankle plantar flexors, although this cannot be confirmed in the present study.

### Sex differences in postural control

We found that females had a greater mean anterior COP position than males (Table 3). This sex difference could be due to anthropometric differences (e.g., weight, height) between males and females rather than sex differences in postural control strategy (or ability), as found or suggested in previous studies for variables reflecting postural performance [34, 46–48]. In the present study, greater height and weight in males compared to females (Table 1) and moderate-to-high negative correlations between anthropometric variables (height and weight) and AP position (but none of the other COP variables; Table 2) were found. Interestingly, when analyzed separately for each sex group, we found significant correlations between AP position and body weight in females for all task conditions (except for FL-EC; Table 2), whereas males showed only one significant correlation between sway area and height during QS-EC, potentially suggesting sex-specific stability behavior related to body factors in young adults [49, 50]. However, this should be interpreted with caution, as we still found a sex difference in the AP position normalized by each of height and weight, possibly indicating other contributors or confounding factors to the sex differences in COP behavior. For example, different foot size/feature (length, width, and arch area/height) between the sex groups (although not measured in the present study) may become another contributor, given the relationship between foot size and AP postural stability (e.g., see [51]).

### Interactions between sex and fatigue on postural control

We found a sex X fatigue interaction for mean COP sway velocity during the most challenging task (FL-EC), where males showed a significant and prolonged increase in sway velocity (~28%) up to 15 min following exercise, whereas females showed a non-significant increase (~2%) and then recovered to a slightly lower value (~5%) than baseline by 5–10 min post-exercise (Fig 4). This is consistent with a recent finding [22], where an increase in COP sway velocity during one-leg standing following a repetitive lifting task was observed in males, while females showed a slight decrease. This result could indicate an increased ankle stiffness strategy with fatigue, but only in males, to maintain stability. Given the similar fatigability (time to task failure) between the sex groups in the present study, the presence of the ankle stiffness strategy could be due to a disadvantage in terms of biomechanical factors (e.g., greater weight/height and related higher COM) for static stability in males compared with females, as reported in a review by Paillard [3]. Amongst potential biomechanical factors, greater body weight in males compared to females may be a primary contributor to the sex X fatigue interaction on COP sway velocity during the most challenging task because the interaction was eliminated with the COP velocity normalized by the weight of the participants.

Given the compensatory movements with ankle plantar flexors fatigue mentioned in the “Fatigue effects on postural control” section above, we speculate that the sex X fatigue interaction suggests the involvement of different postural control strategies with ankle fatigue between females and males, with males potentially relying preferentially on ankle/foot control (possibly relying on non-fatigued ankle invertors, evertors, dorsiflexors, and toe flexors), and females preferentially relying on proximal joint movements (e.g., hip and trunk) to maintain stability. Such a sex difference in control strategy is supported by previously observed greater fatigue-induced ankle torque variability during feet-together standing with EC in males compared to females [24], and a fatigue-induced increase in trunk accelerations (induced by hip movement) accompanying a decrease in COP velocity (ankle control) in females during one-leg standing [52]. However, the sex X fatigue interaction and proposed potential control mechanisms involved should be interpreted with caution as we did not measure ankle/foot and proximal joint movements.

### Interactions between vision and fatigue on postural control

A vision X fatigue interaction was found for mean AP COP position for both QS and FL tasks, which was due to a greater backward shift of the COP during recovery (from 5 to 15 min) for the tasks with EC (significant) compared with EO (not significant) (Fig 2A). This is consistent with previous findings of fatiguing ankle plantar flexors, which showed greater postural sway changes (or impairment) with no-vision and/or disturbed vision compared to normal vision during upright standing on one leg [7, 14, 25, 27] or two legs [26, 53]. This suggests a significant role of visual information for maintaining stability [54, 55], compensating declines in the neuromuscular system and proprioception of fatigued ankle plantar flexors [3].

It was suggested that such vision effects could be dependent on task difficulty. For example, Bisson and colleagues (2010) found greater fatigue-induced COP sway displacement with EC compared to EO during one-leg standing, but not during two-leg standings (semi-tandem and feet together). However, this is not consistent with our findings showing similar fatigue X vision interactions during both QS and FL tasks (although different at 15 min, see Fig 2A). This could be explained by the use of the representation of the COP on a computer screen, which would enhance the role of vision for compensating instability with fatigue and lead to a vision effect regardless of task difficulty (QS vs. FL). This contrasting finding may also be attributed to the use of the relatively close vision target (1.8 m) in the present study compared to the relatively distant one (2.5 m) used by Bisson et al., given the effect of vision target distance on postural control [54]. This is supported by a previous study [53], which found the reduced contribution of vision to compensating fatigue-induced instability with a distant target (4 m) compared to near one (1 m). These findings showcase the importance of task specificity when determining vision effects on postural changes associated with fatigue.

A few limitations should be mentioned for the present study. A motion capture system and electromyographic (EMG) measures were not used to assess body movements and muscle activation to compare compensatory postural strategies (e.g., ankle, hip, and trunk movements) produced by fatigue of ankle plantar flexors between males and females. Vision impairments and correction and ankle stability in participants was not formally assessed in the present study. However, participants who typically wore glasses were asked to wear them for the session, and the absence of known ankle instability was confirmed verbally. Also, we did not obtain information on the phases of the menstrual cycle in female participants, which has been found to affect postural stability [56, 57]. However, this would likely not be a major confounding factor to sex differences in postural control changes with fatigue, given the smaller effect of the menstrual cycle on fatigability compared to other general sex differences in fatigability [17]. Nevertheless, this study provides useful information regarding sex-specific postural control changes with fatigue, which may contribute to improve the design and prescription of stability-demanding physical training or rehabilitation exercise.

## Conclusion

We found a sex X fatigue interaction for mean COP sway velocity during the most challenging task (FL-EO) in healthy young adults, where only males showed a significant increase in sway velocity after fatigue of ankle plantar flexion. This interaction suggests that different postural control strategies may be used with fatigue between sex groups. We also found a significant contribution of vision to AP stability with fatigue regardless of task difficulty (QS vs FL).

## Supporting information

S1 Dataset This data includes COP sway variables which were collected before and immediately after exercise, and throughout the recovery period (2, 5, 10, 15 min). (XLSX)
